# ^89^Zr-nimotuzumab for immunoPET imaging of epidermal growth factor receptor I

**DOI:** 10.18632/oncotarget.24965

**Published:** 2018-03-30

**Authors:** Rufael Chekol, Viswas Raja Solomon, Elahe Alizadeh, Wendy Bernhard, Darrell Fisher, Wayne Hill, Kris Barreto, John Francis DeCoteau, Angel Casaco Parada, Clarence Ronald Geyer, Humphrey Fonge

**Affiliations:** ^1^ Department of Medical Imaging, University of Saskatchewan, College of Medicine, Saskatoon, SK, Canada; ^2^ Saskatchewan Centre for Cyclotron Sciences (SCCS), The Fedoruk Centre, Saskatoon, SK, Canada; ^3^ Department of Pathology and Laboratory Medicine, University of Saskatchewan, College of Medicine, Saskatoon, SK, Canada; ^4^ Versant Medical Physics and Radiation Safety, Boston, MA, USA; ^5^ Centre for Molecular Immunology, Havana, Cuba; ^6^ Department of Medical Imaging, Royal University Hospital Saskatoon, Saskatoon, SK, Canada

**Keywords:** epidermal growth factor receptor I, zirconium-89, nimotuzumab, immunoPET/CT, radiochemistry

## Abstract

**Rationale:**

Epidermal growth factor receptor (EGFR) upregulation is associated with enhanced proliferation and drug resistance in a number of cancers. Nimotuzumab is a humanized monoclonal antibody with high affinity for EGFR. The objective of this study was to determine if ^89^Zr-DFO-nimotuzumab could be suitable for human use as a PET probe for quantifying EGFR *in vivo*.

**Methods:**

To evaluate the pharmacokinetics, biodistribution, microPET imaging, radiation dosimetry, and normal tissue toxicity in tumor and non-tumor bearing mice of ^89^Zr-desferoxamine-nimotuzumab (^89^Zr-DFO-nimotuzumab) of a product prepared under GMP conditions. Nimotuzumab was conjugated to DFO and radiolabeled with ^89^Zr. ^89^Zr-DFO-nimotuzumab was characterized by *in vitro* gel-electrophoresis, biolayer interferometry (BLI) and flow cytometry. ^89^Zr-DFO-nimotuzumab was evaluated *in vivo* by microPET and *ex vivo* by biodistribution in healthy and EGFR-positive tumor bearing mice.

**Results:**

Flow cytometry with A431 cells showed no significant difference in the dissociation constant of nimotuzumab (13 ± 2 nM) compared with DFO-nimotuzumab (17 ± 4 nM). PET imaging in mice xenografts showed persistently high tumor uptake with the highest uptake obtained in DLD-1 xenograft (18.3 %IA/cc) at 168 hp.i. The projected human effective dose was low and was 0.184 mSv/MBq (0.679 rem/mCi) in females and 0.205 mSv/MBq (0.757 rem/mCi) in males. There was no apparent normal tissue toxicity as shown by cell blood counts and blood biochemistry analyses at 168-fold and 25-fold excess of the projected human radioactive and mass dose of the agent.

**Conclusion:**

^89^Zr-DFO-nimotuzumab had low organ absorbed dose and effective dose that makes it suitable for potential human use.

## INTRODUCTION

Overexpression of EGFR is implicated in all aggressive cancers of epithelial origin including squamous cell head & neck (90 – 100%) [1], glioma (90 – 100%) [2], non-small cell lung (75 – 90%), colorectal (80 – 85%) [3], breast (20 – 30%) [4] and cervical [5] cancers. Anti-EGFR antibodies e.g. cetuximab, panitumumab, and nimotuzumab have been used for treating different EGFR-positive cancers. With the exception of nimotuzumab, these antibodies cause significant cutaneous toxicity in 45 - 100% of patients. In contrast, nimotuzumab is better tolerated and has low skin toxicities, because its “affinity optimized” binding characteristics ensures transient binding to low EGFR-expressing healthy tissues such as the skin. Garrido *et al* [6] showed that the low skin toxicity of nimotuzumab is attributed to its transient monovalent binding in low-EGFR expressing tissues such as the skin and gastro-intestinal mucosa (these tissues account for the dose-limiting toxicities observed with anti-EGFR antibody treatments). This low transient monovalent binding is due to a 10-fold lower affinity of nimotuzumab for EGFR compared to cetuximab or panitumumab [6].

Existing *ex vivo* assays for monitoring EGFR expression such as immunohistochemistry (IHC), fluorescence *in situ* hybridization (FISH), and quantitative polymerase chain reaction (qPCR) are invasive and not very reliable [7, 8]. These techniques require frequent biopsies that are impossible for most patients. In addition, biopsy sampling is inherently flawed because of the intra- and inter-lesion heterogeneity of tumors. It is also well known that the EGFR expression changes over time [9]. *In vivo* measurement of the EGFR expression in cancer will offer several advantages over current *ex vivo* methods, including measuring EGFR expression over the entire tumor volume rather than just a part of the tumor, assessing the biologic availability of EGFR *in vivo*, evaluating effects of therapy on EGFR expression, and quantifying EGFR expression of all lesions in real time.

A few ImmunoPET probes have been developed to image EGFR expression *in vivo* [10–14]. ^89^Zr-labeled desferoxamine (DFO) conjugated cetuximab (^89^Zr-DFO-cetuximab) is currently been investigated in clinical trials for PET imaging of EGFR [11, 14]. Menke-van der Houven van Oordt *et al.* administered 37 MBq of ^89^Zr-DFO-cetuximab to 10 colorectal cancer patients with wild-type KRAS mutations two hours after treatment with therapeutic doses of cetuximab. PET scans were acquired at 1 to 10 days post ^89^Zr-DFO-cetuximab injection [14]. 6/10 patients that had positive ^89^Zr lesions showed clinical benefit with cetuximab, while 4/10 patients with no positive ^89^Zr-DFO-cetuximab lesions detected by PET did not show clinical response to cetuximab. The strong positive correlation between ^89^Zr-DFO-cetuximab uptake and clinical response to cetuximab treatment implies that this imaging agent can be used to select patients that would benefit from cetuximab therapy. Because of its low binding to tissues that express low EGFR, such as skin, a nimotuzumab immunoPET agent may be potentially advantageous over ^89^Zr-DFO-cetuximab or ^89^Zr-DFO-panitumumab probes as the low off target binding may provide a more favorable dosimetry and unequivocal delineation of secondary lesions in organs such as the liver that express low EGFR and are frequent metastatic sites for many EGFR positive cancers. Such a favorable dosimetry may also permit repeated tracer injection.

A few probes have been developed using nimotuzumab for imaging EGFR expression by SPECT. Vallis *et al.* evaluated a ^99m^Tc-nimotuzumab in a phase I trial [15]. In this study 12 patients received 999 MBq of ^99m^Tc-nimotuzumab and were imaged at 30 min for up to 24 h post injection. The effective dose of ^99m^Tc-nimotuzumab was 1.34 ± 0.02 × 10^−8^ mSv Bq^−1^. One patient with squamous cell carcinoma of the mouth showed a positive scan. EGFR positivity was not an entry criteria for this trial. PET offers unique advantages such as superior spatial resolution, better signal to-noise ratio and quantitative capabilities over SPECT which makes it the modality of choice for radiolabeled antibodies/fragment. Our goal was to develop a clinical-grade ^89^Zr-DFO-nimotuzumab for imaging EGFR expression using PET. Here, we describe the development and, *in vitro* and *in vivo* characterization of ^89^Zr-DFO-nimotuzumab that will meet all Health Canada guidelines for a phase I clinical study.

## RESULTS

### Conjugation and quality control of DFO-nimotuzumab kit formulation

**Figure 1:**
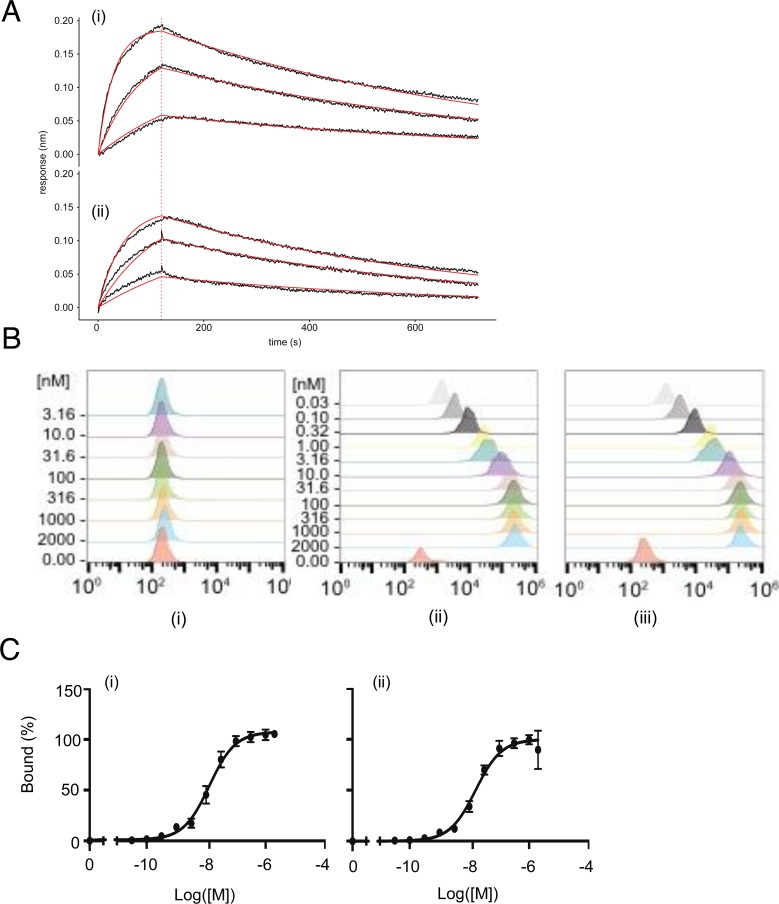
**(A-C)** *In vitro* characterization of DFO-nimotuzumab kit formulation by biolayer interferometry (BLI) and flow cytometry. (A) BLI binding curves for nimotuzumab (i) and DFO-nimotuzumab (ii) at different concentrations (55.5, 166.7 and 500 nM). A K_D_ of 23 ± 3 nM) and 48 ± 9 nM was obtained for nimotuzumab and DFO-nimotuzumab, respectively. Solid black line indicates raw data, solid red line indicates curve fit, vertical dashed red line indicates transition from association to dissociation phase. (B) Flow cytometry results of nimotuzumab in control MDA-MB-435 (i) with very low levels of EGFR and corresponding EGFR-positive A431 cells (ii and iii). No substantial binding was seen in MDA-MB-435 cells. Binding of DFO-nimotuzumab (iii) in A431 cells was comparable with nimotuzumab (ii). (C) Nimotuzumab (i) and DFO-nimotuzumab (ii) showed saturation binding by flow cytometry with a K_D_ of 13 ± 2 nM and 17 ± 4 nM, respectively.

The conjugation of *p*-Bz-SCN-DFO to nimotuzumab resulted in an average of two DFO molecules per antibody molecule as determined by the difference in molecular weight of nimotuzumab and DFO-nimotuzumab as measured by bioanalyzer. Nimotuzumab and DFO-nimotuzumab were >95 % pure on HPLC with <2 % aggregates in the latter. Following conjugation, the resulting DFO-nimotuzumab kit was characterized for stability, binding to recombinant EGFR, aggregation, and size. In stability studies, samples that were stored at 4°C showed < 4% degradation while no degradation was seen with samples stored at −80°C for over 6 months. Bioanalyzer confirms that nimotuzumab and DFO-nimotuzumab were >95 % pure with molecular weights of 161.5 kDa and 162.7 ± 0.9 kDa, respectively (Supplementary Figure 1A–1B). The conjugation of DFO to nimotuzumab had a modest but significant impact on the binding to recombinant EGFR by BLI (Figure 1A). The K_D_ was significantly different (p < 0.0001) between nimotuzumab (23 ± 3 nM) and DFO-nimotuzumab (30 ± 1 nM). There was no difference in the association rate (k_on_), however, there was a 1.3-fold increase (*p* < 0.0001) in the dissociation rate of DFO-nimotuzumab (2.0 × 10^−3^ ± 0.8 × 10^−4^ s-1) compared with nimotuzumab (1.5 × 10^−3^ ± 2 × 10^−4^ s-1). *In vitro* binding of nimotuzumab and DFO-nimotuzumab to high EGFR expressing A431 cell line was determined using flow cytometry. There was no significant difference in receptor binding of DFO-nimotuzumab (K_D_ 17 ± 2 nM: Figure 1B, iii and Figure 1C, ii) compared with nimotuzumab (K_D_ 13 ± 2 nM: Figure 1B, ii and Figure 1C, i). DFO-nimotuzumab did not bind control MDA-MB-435 cell line (Figure 1B, i). Stability data obtained by HPLC were identical to those obtained using the bioanalyzer (Supplementary Figure 1C). Similarly, stability studies showed no change in binding affinity by biolayer interferometry (BLI) over the studied period.

### Radiolabelling and characterization of ^89^Zr-DFO-nimotuzumab

The optimal condition to obtain quantitative labeling yield was pH 7 ± 0.2, temperature of 37°C and two hr incubation. To obtain quantitative radiolabeling yield without the need to purify before drug administration, a range of specific activities were tested. The labeling efficiency of ^89^Zr-DFO-nimotuzumab was 91 ± 1.2 % with a specific activity of 0.5 MBq/μg (Table 1). High radiochemical yield of 94 ± 0.9% was obtained albeit at a lower specific activity (0.1 MBq/μg). A radiochemical purity of >98 % was obtained after purification. To investigate product shelf-life, ^89^Zr-DFO-nimotuzumab was analyzed at different time periods using HPLC, following storage at 4°C or room temperature (RT). Starting with 94 % pure ^89^Zr-DFO-nimotuzumab, we obtained >90 % radiochemical purity after 24 h storage at room temperature (RT) (Supplementary Figure 2 and Supplementary Table 1). Loss of ^89^Zr was slightly higher at RT than at 4°C. A maximum shelf-life of 24 h is recommended at 4°C following preparation. Additionally, ^89^Zr-DFO-nimotuzumab was found to transchelate in human plasma following incubation with over 20 % of the radiolabel lost at 72 h post incubation. The pharmaceutical specification of the final ^89^Zr-DFO-nimotuzumab formulation is summarized in Table 2.

**Table 1:** Labeling efficiency and specific activity of DFO-nimotuzumab kit formulation

Amount of antibody (μg) per 10 MBq	Labeling efficiency % ± SD	Specific activity (MBq/μg)
5	21 ± 1.1	2.0
10	86 ± 1.0	1.0
20	91 ± 1.2	0.5
50	92 ± 0.4	0.2
100	94 ± 0.9	0.1

**Table 2:** Radiopharmaceutical analyses shows the formulation meets USP specifications

Analysis	Specification
Bubble point filter membrane integrity	>= 50 psi
Visual appearance	Clear colorless solution, no visible particulate matter.
pH testing	6.8 – 7.4
Strength (mCi/mL)	1 – 5 mCi in 10 mL
Radiochemical purity	iTLC >= 95%
Bacterial endotoxin	<= 175 EU/V
Specific activity	>= 0.2 mCi/mg
Sterility	Sterile (no visible growth)
Environmental profile	<= 1 cfu/plate (contact)
	<= 3 cfu/plate (settling)
	<= 3 cfu/plate (finger)
Immunoreactivity	>= 73%

**Figure 2:**
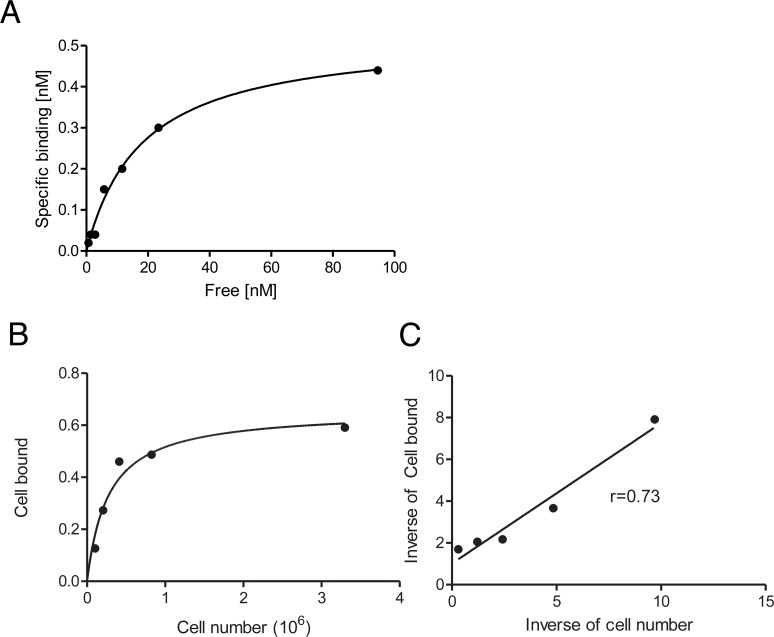
**(A–C)** Radioligand binding assay and immunoreactive fraction determination of ^89^Zr-DFO-nimotuzumab in EGFR-positive DLD-1 cells. (A) Radioligand binding assay ^89^Zr-DFO-nimotuzumab showed a K_D_ of 14 ± 2.4 nM with a ~ 577,000 EGFR/cell. (B & C) The immunoreactive fraction of ^89^Zr-DFO-nimotuzumab was 0.73.

^89^Zr-DFO-nimotuzumab had a K_D_ of 14.05 ± 2.56 nM in EGFR-positive DLD-1 cells (~600,000 EGFR/cell) in a saturation binding analysis (Figure 2), which was similar to the binding constant obtained with A431 by flow cytometry. The immunoreactive fraction of ^89^Zr-DFO-nimotuzumab with DLD-1 cells was 73% (Figure 2B, 2C).

### Pharmacokinetics, biodistribution and radiation dosimetry estimation

**Figure 3:**
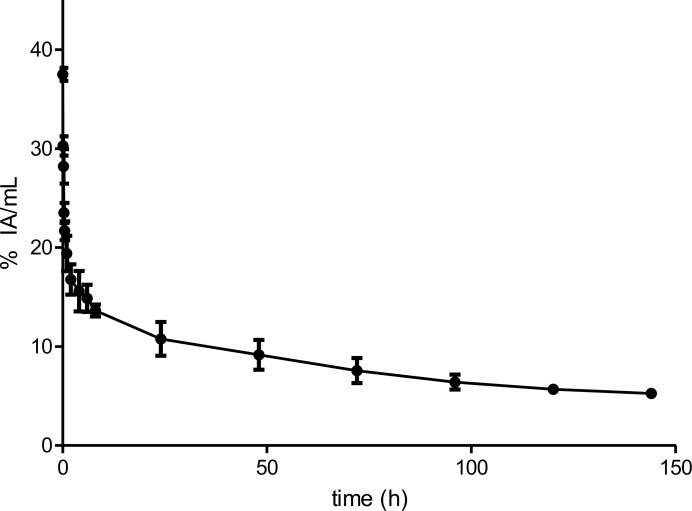
Pharmacokinetics of 89Zr-DFO-nimotuzumab in normal balb-C mice ^89^Zr-DFO-nimotuzumab exhibits a biphasic kinetics with distribution and elimination half-lives of 1.3 (t_1/2α_) and 127.1 (t_1/2β_), respectively. The volume of distribution of the central compartment (V1) was 3.2±0.3 mL mL (107 mL/kg) and the volume of distribution at steady state (Vss) was 8.6±3.4 mL (287 mL/kg). The V1 and Vss volumes correspond to 7.5 L and 20.1 L respectively, in a 70 kg standard adult female. The systemic clearance (CLs) was 0.045±0.0020 mL/h (1.5 mL/h/kg). The CLs values in mice would correspond to 105 mL/h in a 70 kg adult female.

^89^Zr-DFO-nimotuzumab exhibited a bi-phasic half-life with a fast (distribution) t_1/2α_ of 1.3 h and a slow clearance t_1/2β_ of 127.1 h (Figure 3). Biodistribution was measured in normal female (Supplementary Figure 3A) and male (Supplementary Figure 3B) mice. At early time points (up to 72 hp.i), there was significantly higher uptake of the tracer in bones and skin of female mice compared to male mice (Supplementary Figure 3A and 3B). However, uptake in both organs decreased to background levels at 168 hp.i. Other organs did not show differences between male and female mice. The liver had the longest residence time being the principal route of excretion of the tracer. Interestingly, the residence time in the bone marrow and skin were very short indicative of minimal transchelation of the radiometal and low skin binding, respectively (Supplementary Table 2). The liver received the highest dose (female 1.82 cGy/mCi vs male 2.64 cGy.mCi), followed by gall bladder wall (female 1.1 cGy/mCi vs male 1.3 cGy/mCi) and osteogenic cells (female 0.9 cGy/mCi vs male 0.92 cGy/mCi) (Table 3). The projected human effective dose was 0.679 rem/mCi (0.184 mSv/MBq) and 0.757 rem/mCi (0.205 mSv/MBq) in females and males, respectively.

**Table 3:** Projected absorbed organ doses of ^89^Zr-DFO-nimotuzumab

Organ	Female – ^89^Zr absorbed dose (cGy/mCi)	Male ^89^Zr absorbed dose (cGy/mCi)
Adrenals	0.672	0.821
Brain	0.205	0.234
Breasts	0.573	0.601
Gallbladder Wall	1.07	1.30
Lower Large Intestine	0.757	0.779
Small Intestine	0.929	0.954
Stomach	0.693	0.783
Upper Large Intestine	0.882	0.936
Heart Wall	0.601	0.683
Kidneys	0.603	0.775
Liver	1.82	2.64
Lungs	0.481	0.557
Muscle	0.485	0.513
Ovaries	0.656	
Pancreas	0.636	0.819
Red Marrow	0.59	0.624
Osteogenic Cells	0.895	0.918
Skin	0.445	0.462
Spleen	0.431	1.93
Testes		0.402
Thymus	0.452	0.473
Thyroid	0.618	0.623
Urinary Bladder	0.639	0.643
Uterus	0.564	
Total Body	0.624	0.677
Effective dose (rem/mCi)	0.679	0.757

### Toxicity of ^89^Zr-DFO-nimotuzumab in mice

Single dose acute (Day-2) and delayed (Day-14) toxicity was determined in normal Balb-C mice. At Day-2 p.i., there was no significant difference (*p* >0.05) in any of the CBC or blood chemistry parameters in females and males compared to control (saline injected) group (Table 4A). Similarly, there was no significant difference (*p* >0.05) in the treated compared to control group at Day-14 p.i. (Table 4B). Histopathological examination of necropsy stained slices showed no damage to the organs following administration of 168-fold (radioactivity) and 25-fold (mass dose) excess of the projected human dose (data not shown).

**Table 4A:** Acute (2-day) toxicity of ^89^Zr-DFO-nimotuzumab

Parameters	Male ± Std Dev.^*^		Female ± Std Dev.^**^	
Clinical chemistry	Control treated	^89^Zr-DFO-nimotuzumab	Control treated	^89^Zr-DFO-nimotuzumab
Albumin:Globulin (g/L)	1.61 ± 0.13	1.51 ± 0.05	2.08 ± 0.13	2.01 ± 0.22
Albumin (g/L)	29.21 ± 1.09	29.0 ± 0.70	32.8 ± 1.48	32.0 ± 1.41
Globulin (g/L)	18.2 ± 0.84	19.2 ± 0.44	15.8 ± 1.09	16.0 ± 1.22
Alkaline Phosphatase (U/L)	146.41 ± 11.39	139.60 ± 9.91	147.20 ± 6.38	140.80 ± 2.59
ALT (U/L)	24.21 ± 2.58	25.81 ± 1.3	25.80 ± 5.76	26.60 ± 4.51
AST (U/L)	69.81 ± 9.85	83.21 ± 17.98	116.4 ± 82.50	73.0 ± 15.02
Bicarbonate (mmol/L)	17.01 ± 0.7	17.41 ± 1.14	15.4 ± 2.97	18.0 ± 0.70
Ca^2+^ (mmol/L)	2.48 ± 0.07	2.39 ± 0.14	2.28 ± 0.04	2.37 ± 0.13
Cl^−1^ (mmol/L)	109.20 ± 1.483	108.2 ± 0.83	106.0 ± 4.12	108.0 ± 2.12
Creatinine	14.6 ± 1.67	12.8 ± 2.59	18.0 ± 5.43	14.41 ± 3.05
Glucose (mmol/L)	13.68 ± 1.70	12.68 ± 1.29	14.02 ± 1.72	12.50 ± 1.11
K+ (mmol/L)	5.12 ± 0.55	6.48 ± 0.69	5.12 ± 0.85	5.1 ± 0.73
Na+ (mmol/L)	147.8 ± 1.79	145.0 ± 1.58	142.20 ± 4.44	144.2 ± 1.92
Na+/Ka+	29.41 ± 2.9	22.4 ± 2.4	28.40 ± 4.80	28.8 ± 4.87
Total bilirubin (μmol/L)	1.28 ± 0.39	1.42 ± 0.39	0.36 ± 0.33	0.96 ± 0.15
Total protein (g/L)	47.4 ± 0.54	48.2 ± 0.83	48.6 ± 2.19	48.0 ± 1.4
Urea (mmol/L)	6.86 ± 0.6	7.30 ± 0.89	6.86 ± 0.6	7.30 ± 0.88
Cell Blood Counts				
HCT (L/L)	0.47 ± 0.05	0.47 ± 0.02	0.48 ± 0.02	0.48 ± 0.01
HGB (g/L)	144.0 ± 12.73	144.2 ± 5.77	148.20 ± 3.96	150.40 ± 3.05
MCH (pg)	15.05 ± 0.19	14.9 ± 0.22	15.21 ± 0.10	15.18 ± 0.16
MCHC (g/L)	307.3 ± 4.99	307.6 ± 3.8	309.80 ± 3.63	315.0 ± 5.29
MCV (fL)	48.93 ± 0.3	48.42 ± 0.44	48.96 ± 0.48	48.14 ± 0.42
RBC (x10^12^/L)	9.55 ± 0.92	9.68 ± 0.37	9.76 ± 0.3	9.92 ± 0.23
RDW (%)	14.35 ± 0.47	14.26 ± 0.47	14.98 ± 0.41	14.90 ± 0.32
WBC (x10^9^/L)	2.45 ± 1.59	2.9 ± 1.50	3.06 ± 0.90	2.08 ± 0.64

^*^ (male) and ^**^ (female): There were no significant differences in all parameters between control treated and ^89^Zr-DFO-nimotuzumab treated mice following injection of excess of ^89^Zr-DFO-nimotuzumab.

**Table 4B:** Acute (14-day) toxicity of ^89^Zr-DFO-nimotuzumab

Parameters	Male ± Std Dev.		Female ± Std Dev.	
Clinical chemistry	Control treated	^89^Zr-DFO-nimotuzumab	Control treated	^89^Zr-DFO-nimotuzumab
Albumin:Globulin (g/L)	1.37 ± 0.23	1.49 ± 0.07	2.19 ± 0.21	1.94 ± 0.15
Albumin (g/L)	27.40 ± 3.36	29.0 ± 1.41	31.80 ± 0.84	31.40 ± 28.43
Globulin (g/L)	20.21 ± 1.64	19.4 ± 0.89	14.60 ± 1.14	16.2 ± 0.83
Alkaline Phosphatase (U/L)	106.60 ± 11.72	108.2 ± 4.42	127.60 ± 2.30	115.6 ± 4.61
ALT (U/L)	35.4 ± 8.79	27.40 ± 1.14	26.20 ± 3.56	23.60 ± 2.70
AST (U/L)	233.4 ± 143.3	71.0 ± 11.29	78.80 ± 12.48	103.80 ± 28.43
Bicarbonate (mmol/L)	15.81 ± 2.49	18.4 ± 1.51	15.21 ± 1.48	16.01 ± 1.58
Ca^2+^ (mmol/L)	2.35 ± 0.13	2.36 ± 0.10	2.27 ± 0.09	2.27 ± 0.03
Cl^−1^ (mmol/L)	104.6 ± 3.36	106.6 ± 1.52	107.2 ± 2.58	107.0 ± 1.58
Creatinine	13.4 ± 1.82	14.60 ± 2.70	12.8 ± 0.84	13.8 ± 2.0
Glucose (mmol/L)	12.22 ± 1.37	11.89 ± 0.61	11.42 ± 0.85	11.62 ± 2.27
K+ (mmol/L)	6.20 ± 1.06	5.18 ± 0.33	4.52 ± 0.47	4.30 ± 0.50
Na+ (mmol/L)	147.40 ± 4.39	149.6 ± 1.52	148.0 ± 1.87	147.4 ± 0.89
Na+/Ka+	24.40 ± 5.03	29.0 ± 2.0	32.81 ± 4.03	34.80 ± 3.7
Total bilirubin (μmol/L)	1.32 ± 0.33	0.62 ± 0.24	0.82 ± 0.18	1.02 ± 0.33
Total protein (g/L)	47.6 ± 2.4	48.4 ± 1.95	46.41 ± 1.14	47.60 ± 0.54
Urea (mmol/L)	7.48 ± 0.39	9.52 ± 0.93	7.48 ± 0.39	9.52 ± 0.93
Cell Blood Counts				
HCT (L/L)	0.46 ± 0.02	0.47 ± 0.03	0.47 ± 0.01	0.47 ± 0.01
HGB (g/L)	147.40 ± 5.22	148.3 ± 6.75	148.60 ± 4.04	150.60 ± 4.34
MCH (pg)	15.04 ± 0.20	15.35 ± 0.13	15.30 ± 0.14	15.66 ± 0.27
MCHC (g/L)	317.40 ± 5.68	311.30 ± 7.6	313.80 ± 5.93	318.4 ± 6.22
MCV (fL)	47.46 ± 0.41	49.33 ± 1.68	48.76 ± 0.65	49.18 ± 0.59
RBC (x10^12^/L)	9.8 ± 0.41	9.66 ± 0.49	9.71 ± 0.25	9.61 ± 0.33
RDW (%)	13.66 ± 0.11	14.73 ± 0.29	14.24 ± 0.25	14.64 ± 0.25
WBC (x10^9^/L)	1.92 ± 1.22	2.18 ± 0.71	2.14 ± 1.12	1.25 ± 0.06

^*^ (male) and ^**^ (female): There were no significant differences in all parameters between control treated and ^89^Z-DFO-nimotuzumab treated mice following injection of excess of ^89^Zr-DFO-nimotuzumab.

### Biodistribution and microPET in tumor bearing mice

**Figure 4:**
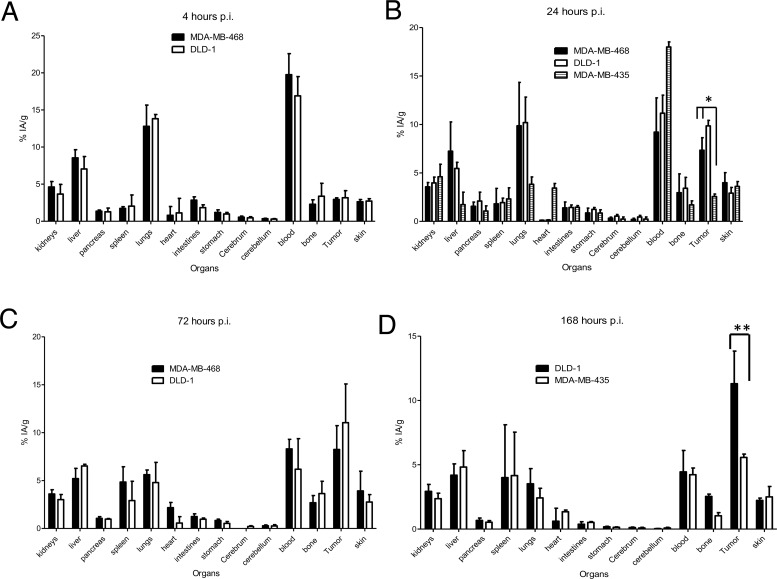
**(A–D)** Biodistribution of ^89^Zr-DFO-nimotuzumab in EGFR-positive MDA-MB-468, DLD-1, and control (very low EGFR expressing) MDA-MB-435 xenografts at 4, 24, 72 and 168 h post injection. Athymic CD-1 nude mice xenografts were injected via a tail vein with 10 MBq 10 μg of ^89^Zr-DFO-nimotuzumab followed by biodistribution studies. Uptake in MDA-MB-468 and DLD-1 tumors was significantly higher than in control MDA-MB-435 at 24 (^*^ *p* < 0.05) and 168 h (^**^ *p* < 0.05) post injection.

^89^Zr-DFO-nimotuzumab biodistribution was analyzed at 4, 24, 72 and 168 hp.i (Figure 4A–4D) in mice bearing xenografts with medium EGFR expression (DLD-1, MDA-MB-468) and a control negative for EGFR expression (MDA-MB-435). Tumor uptake (expressed as % injected activity per gram (% IA/g) or % IA per volume (% IA/cc)) in DLD-1 was slightly higher than for MDA-MB-468 at 24 h (9.85 ± 0.57 vs 7.30 ± 1.29% IA/g) and 72 hp.i (11.04 ± 4.04 vs 8.24 ± 2.49 % IA/g) even though this was not significant. Control xenograft had significantly lower tumor uptake (2.56 ± 0.26 % IA/g) at 24 hp.i. There was a high lung uptake (DLD-1: 13.82 ± 0.57 % IA/g; MBDA-MB-468: 12.80 ± 2.86 % IA/g) of the tracer at early time point (4 hp.i). However, this decreased to 4.79 ± 2.1 % IA/g (DLD-1) and 5.61 ± 0.49 % IA/g (MDA-MB-468) at 72 hp.i. There was also a slight increase in bone uptake of the tracer over time which is indicative of some transchelation and uptake of bone seeking free ^89^Zr^4+^. The highest bone uptake observed at 72 h was 3.64 ± 1.28 % IA/g. Tumor to blood ratio for DLD-1 and MDA-MB-468 was 0.15 and 0.18, respectively at 4 hp.i but this increased to 1.0 and 1.79 respectively at 72 hp.i. Highest tumor-to-blood ratio was 2.54 at 168 hp.i for DLD-1 tumor.

**Figure 5:**
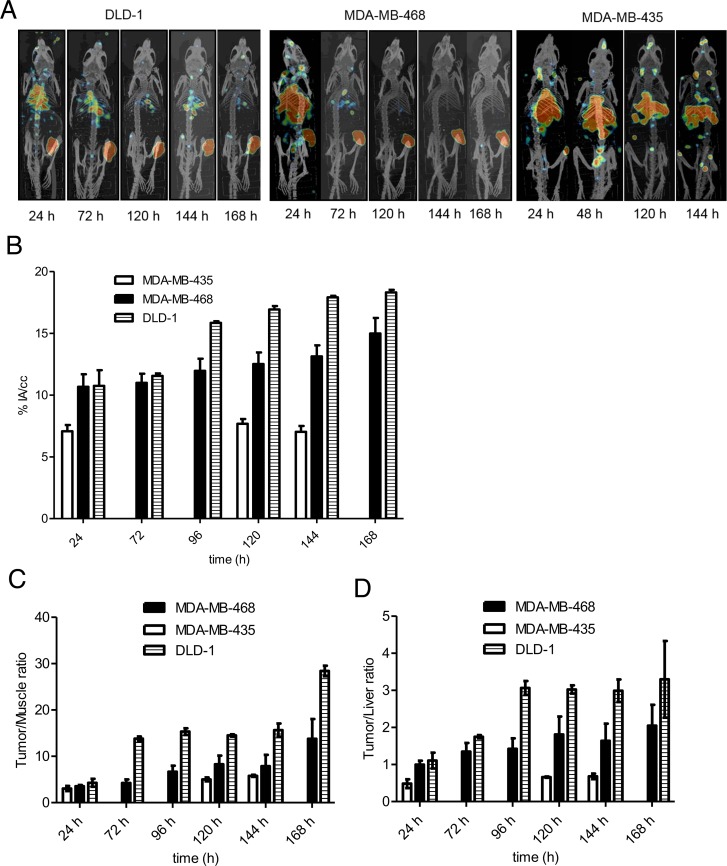
**(A–D)** PET/CT imaging and image analyses in mice xenografts. (A) Maximum intensity projection (MIP) PET/CT images of a representative mouse bearing EGFR-positive DLD-1 and MDA-MB-468, and control MDA-MB-435 xenografts at different time points post ^89^Zr-DFO-nimotuzumab injection. Xenografts are located in right thigh of the hind leg. (B) Except at 24 h post injection, significantly higher tumor uptake was seen in EGFR xenografts compared with control xenograft. (C) Tumor/muscle and (D) Tumor/liver ratios in same mice groups.

PET imaging showed persistently high tumor uptake in EGFR-positive xenografts as seen in the maximum intensity projection images of these mice at 24 – 168 hp.i (Figure 5A).^89^Zr-DFO-nimotuzumab uptake on PET was expressed as % IA/cc (Figure 5B). There was no significant difference (*p* > 0.05) in uptake between the three EGFR-positive xenografts at 24 hp.i. At 24 hp.i uptake in DLD-1 (10.7 ± 0.4 % IA/cc) and MDA-MB-468 (10.9 ± 1.3 % IA/cc) were significantly higher (p = 0.001) than in control MDA-MB-435 (7.07 ± 0.9 % IA/cc). At 96 and 120 hp.i, DLD-1 had the highest uptake (p < 0.0001) compared with MDA-MB-468 xenografts. There was increasing uptake in DLD-1 and MDA-MB-468 xenografts for up to 168 hp.i. The highest tumor-to-background (muscle) ratio was observed at 168 hp.i for DLD-1 (28.4 ± 1.1) and this was significantly higher (p < 0.01) than for MDA-MB-468 (13.8 ± 4.2) (Figure 5C). There were significant differences in tumor/muscle ratios between DLD-1 and control MDA-MB-435 at other time points. Tumor/muscle ratios were higher for MDA-MB-468 compared with control MDA-MB-435 although these differences were not significant. Tumor/liver ratios were significantly higher in DLD-1 and MDA-MB-468 compared with control MDA-MB435 at all time points (Figure 5D).

## DISCUSSION

EGFR is frequently overexpressed in most cancers of epithelial origin where it promotes enhanced proliferation and confers resistance to targeted therapies. Existing methods to monitor EGFR expression have yielded sub-optimal results. In addition, the expression of EGFR changes over time. It is important to develop a non-invasive method for EGFR quantitation *in vivo*. Nimotuzumab is an affinity-optimized antibody that is approved for squamous cell carcinoma of head & neck, glioma, pancreatic and nasopharyngeal cancers in many jurisdictions. Other radiolabeled nimotuzumab radioimmunoconjugates including ^111^In-nimotuzumab and ^99m^Tc-nimotuzumab have been evaluated previously in preclinical and early-phase clinical trials [10, 15]. While SPECT agents labeled with ^111^In and ^99m^Tc may offer some advantages over other PET isotopes such as lower radiation dose exposure, the inherent image quality and quantitative capabilities of PET is superior to SPECT.

Here, we evaluated the PET imaging properties of ^89^Zr-DFO-nimotuzumab. First, we developed a kit preparation of DFO-nimotuzumab in accordance with USP standards. Several batches of DFO-conjugated nimotuzumab have been produced to date and all have met and exceeded USP specifications (Table 2). Importantly, the conjugation of DFO did not significantly alter the binding of nimotuzumab to EGFR-positive cells and the shelf-life of DFO-nimotuzumab was 6 months at 4°C and even longer at −80°C.

^89^Zr-DFO-nimotuzumab did not cause any changes in CBC and blood chemistry following injection of excess mass and radioactivity dose of the projected human dose of the tracer – 74 MBq 10 mg. The projected effective dose in humans was 0.679 rem/mCi (0.184 mSv/MBq in female and 0.757 rem/mCi (0.205 mSv/MBq) in males. There are several immunoPET imaging agents in clinical trial, notably, anti-Her2 ^89^Zr-DFO-trastuzumab and anti-EGFR ^89^Zr-DFO-cetuximab are in advanced development. The effective dose of ^89^Zr-DFO-trastuzumab as determined by Laforest *et al*. was 0.61 mSv/MBq. Similarly, Bhattacharyya *et al*. determined the effective dose of ^89^Zr-DFO-panitumumab was 0.57 mSv/mCi. The effective dose ^89^Zr-DFO-nimotuzumab was 3.3-fold and 3.1-fold lower than for ^89^Zr-DFO-trastuzumab and ^89^Zr-DFO-panitumumab, respectively [16, 17]. The lower absorbed dose of ^89^Zr-DFO-nimotuzumab to organs offers the possibility of administering multiple doses and remain within the lower limit of organ dose set by the FDA. Radioactive Drug Research Committee (FDA RDRC) guidelines, which stipulates that for whole body, active blood-forming organs, lens of eye, and gonads, the single dose must not exceed 30 mCv (3 rems) or annual dose of 50 mSv (5 rems). In line with the FDA RDRC guidelines, patients on ^89^Zr-DFO-trastuzumab and ^89^Zr-DFO-cetuximab clinical trials were infused an average dose of 37 MBq. A 37 MBq dose corresponds to a 22.57 mSv and 21.1 mSv effective dose for ^89^Zr-trastuzumab and ^89^Zr-cetuximab, respectively. However, administration of 37 MBq of ^89^Zr-nimotuzumab will result in an estimated dose of only 6.81 mSv. By comparison, the estimated dose of ^18^F-FDG, the most widely used PET imaging agent is 7.4 mSv for an adult patient receiving 370 – 540 MBq [18]. Assuming the same (37 MBq) patient dose as was infused for the other immunoPET agents the administration of ^89^Zr-DFO-nimotuzumab would result in scans of diagnostic quality that offers a similar or lower dose than FDG. Similar to FDG, the low body dose to patients of ^89^Zr-DFO-nimotuzumab could allow for repeated dosing or follow up scans that cannot be done with other immunoPET agents such as ^89^Zr-DFO-cetuximab. This low observed toxicity was confirmed by CBC and blood biochemistry studies which showed no significant difference in all blood cells and chemistry parameters investigated.

^89^Zr-DFO-nimotuzumab showed early high uptake in tumor xenografts. Tumor uptake was up to 6.2% IA/cc at 4 hp.i and this uptake increased and remain persistently high at the end of imaging 168 hp.i where the highest uptake was observed in DLD-1 xenograft (18.3 %IA/cc). Tumors were clearly delineated at 24 hp.i with the highest tumor to muscle ratios observed at 168 hp.i. Early delineation of tumor may be possible in patients with ^89^Zr-DFO-nimotuzumab given the early high tumor uptake and the rapid off-target clearance of the tracer (as evidence from the lower organ doses). IgGs have not been the molecule of choice for ImmunoPET because of the slow accumulation, slow blood clearance, and high background. As a result, images of diagnostic quality have mostly been obtained at 4 – 7 days p.i.. In the Even *et al.* study, the recommended imaging time using ^89^Zr-DFO-cetuximab for patients with colorectal cancer was 6 – 7 day p.i. [19]. Similarly, in the study by Dijkers *et al*. the best time for assessment of ^89^Zr-trastuzumab uptake by tumors was 4–5 days [20].

We developed a clinical-grade ^89^Zr-DFO-nimotuzumab as a potential immunoPET agent for imaging of patients with EGFR-positive cancers. The kit formulation DFO-nimotuzumab and imaging agent ^89^Zr-DFO-nimotuzumab met the USP and Health Canada QC requirements for an investigational agent. The projected human effective dose is more than three-fold lower than for similar immunoPET imaging agents in clinical trials. At a human dose of 37 MBq (10 mg) the effective dose for ^89^Zr-DFO-nimotuzumab would be comparable to that of ^18^F-FDG. Our results showed that ^89^Zr-DFO-nimotuzumab could provide earlier tumor delineation and with low organ radiation dose.

## MATERIALS AND METHODS

The conjugation of nimotuzumab to DFO and labeling with ^89^Zr were performed following good manufacturing practice (GMP) conditions at the Saskatchewan Centre for Cyclotron Sciences (SCCS). Materials and solvents for the labeling procedure were sterile and endotoxin and metal free except for Amicon Ultra-4 Centrifugal Filter 30K NMCO, (EMD Millipore). Protein concentration, endotoxin content, and sterility were measured. All chemicals used for the study complied with U.S. Pharmacopoeia specifications. Clinical-grade nimotuzumab was obtained from the Center for Molecular Immunology (CIM: Havana) and was used as per terms of the research collaboration and material transfer.

### Cell lines and xenografts

Human cancer cell lines MDA-MB-468, DLD-1, and A431 that express EGFR and negative control (no EGFR expression) MDA-MB-435 cell line were obtained from ATCC (Rockville, MD, USA). Cells were propagated by serial passage in MEM/EBSS medium, supplemented with 10 % fetal bovine serum (Biochrom) at 37°C in a humidified atmosphere of 5 % CO2.

All animals used in imaging experiments were cared for and maintained under the supervision and guidelines of the University of Saskatchewan Animal Care Committee (protocol # 20160005). Female CD-1 nude mice were obtained from Charles River Canada (St-Constant, Quebec) at 4 weeks of age and housed in a 12 h light, 12 h dark cycle in a temperature and humidity controlled vivarium. Animals had libitum access to mouse diet (Lab Diet, St. Louis, Missouri) and water. After one week of acclimatization, mice were subcutaneously injected with a suspension of 5 – 10 × 10^6^ MDA-MB-468, DLD-1, A431 or MDA-MB-435 cells in 100 μL of a 1:1 mixture of serum-free MEM/EBSS medium (HyClone Laboratories, Logan, Utah) and Matrigel matrix basement membrane (Discovery Laboware, Inc. Bedford, MA) at the right? hind limb of each mouse. Tumor growth was followed with caliper measurements.

### Conjugation of nimotuzumab with p-SCN-Bz-desferoxamine

Nimotuzumab (5 mg/mL) in PBS was buffer exchanged in 0.1 M NaHCO_3_ (pH 9) using centrifugal filters (Amicon Ultra-4 Centrifugal Filter 30K NMCO, EMD Millipore) and concentrated to 10 mg/mL nimotuzumab in the bicarbonate solution [21]. A ten-fold mole excess of p-SCN-Bz-Deferoxamine (DFO: Macrocyclics) in DMSO was added dropwise to the antibody solution (final volume of DMSO was kept below 5 %). The final concentration of the reaction mixture was 9.5 mg/mL. The reaction mixture was incubated at 37°C on a shaker at 650 RPM for an hour. The reaction mixture was cooled to room temperature and the unreacted DFO was removed by two consecutive centrifugations with desalting columns (Zeba™ Spin Desalting Columns, 7K MWCO, Thermofisher). The buffer was exchanged with PBS using centrifugation with centrifugal filters and sterilized with 0.2 μm hydrophilic PTFE membrane filters (Ultrafree®-CL Centrifugal filters, Millipore). The solution of DFO-nimotuzumab was aliquoted and stored at −80°C until further use.

### Quality control of DFO-nimotuzumab

Nimotuzumab and DFO-nimotuzumab purity was determined using SEC-HPLC (Waters 2796 Bioseparations Module, Waters 2487 Dual λ Absorbance Detector, XBridge® BEH 200A SEC 3.5 μm 7.8 × 300 mm column, Waters Corporation). The UV-Detector was set at 220 and 280 nm and the solvent system was PBS at a flow rate of 0.6 mL/min.

The analysis of MW and purity of conjugated samples were performed on an Agilent 2100 Bioanalyzer using Agilent High Sensitivity Protein 250 Kit) using manufacturer's protocol. The ladder or antibody solution in non-reducing buffer was heated to 95^°^C for 5 minutes before analysis. The size and relative peak area were calculated using Agilent 2100 Expert software. The difference in molecular weight between the nimotuzumab and DFO-nimotuzumab was used to determine the average number of chelator (DFO) on the antibody.

Binding kinetics between the antibodies and EGFR were measured using BLI with ForteBio Octet RED384 (PALL Corporation). Antibodies were immobilized on Anti-human FAB-CH1 sensors (18-5104, Forte Bio) according to manufactures instructions. The equilibrium dissociation constant (K_D_) was obtained using a 1 to 1 binding model with global fitting. Data analysis and curve fitting was performed using Data Analysis software 7.1.0.33 (Forte Bio).

The sterility of nimotuzumab and DFO-nimotuzumab was measured according to USP 71 and bacterial endotoxin test, according to USP 85. Thioglycollate, Trypone Soy Broth, Tryptic Soy Agar (TS) media were used as culture media. For endotoxin limit test, the FDA approved Endosafe-Portable Test System (Charles River Laboratories) was used.

### *In vitro* binding assay by flow cytometery

A431 cells were collected and washed with PBS + 2 % FBS. nimotuzumab and DFO-nimotuzumab were titrated at a minimum of a 10-fold excess onto cells (twelve concentration points starting from 2 μM). Cells with nimotuzumab and DFO-nimotuzumab were incubated for 30 minutes at room temperature followed by 15 minutes on ice. Cells were washed and suspended in a 1:50 dilution of FITC labelled Goat F(ab')2 fragment anti-human IgG (H + L) antibody (Beckman Coulter) and incubated for 30 minutes on ice in the dark. Cells were washed and suspended in PBS + 2 % FBS and analyzed using a Gallios flow cytometer (Beckman Coulter) on the FL1 channel.

### ^89^Zr radiolabeling of DFO-nimotuzumab

1 M Hepes pH 7.4 (Fisher Scientific) was added to ^89^Zr in oxalic acid (Washington University School of Medicine in St. Louise) and 2 M NaCO_3_ (pH 11) was added drop wise while measuring the pH with pH paper until the oxalic acid was neutralized (pH 7 ± 0.2). DFO-nimotuzumab was then added to the ^89^Zr solution at five different specific activities (2, 1, 0.5, 0.2 and 0.1 MBq/μg). The reaction mixture was incubated at 37°C on a shaker at 650 RPM for two hours. The reaction mixture was cooled to room temperature and an aliquot was injected onto SEC-HPLC (the same HPLC and solvent system as above) for radiochemical quality control and radiolabeling efficiency determination. The eluate from the SEC-HPLC was collected to determine the recovery of radioactivity. Oxalate, hepes and other impurities (less than 30 kDa) were removed from crude reaction mixture by single centrifugation in spin columns (Amicon Ultra-4 Centrifugal Filter 30K NMCO, 4 mL, EMD Millipore) and sterilized by filtration with 0.2 μm hydrophilic PTFE membrane filter (Ultrafree®-CL Centrifugal filters 4 mL, Millipore). The final solution was formulated in PBS. The chemical and radiochemical purity of final solution was determined by SEC-HPLC. ^89^Zr-DFO-nimotuzmab with a radiochemical purity of more than 90% was used for *in vitro* and *in vivo* experiments.

The storage stability (shell life) of nimotuzumab and DFO-nimotuzumab at −80°C and 4°C (quadruplets for each) was determined by analyzing aliquots from each sample by SEC-HPLC. An aliquot was thawed from −80°C on a monthly basis and analyzed by HPLC to determine the purities of the solution over time compared to the initial purities of the immunoconjugates. The stability of ^89^Zr-DFO-nimotuzumab was evaluated in solution (0.9% NaCl) and in human plasma at 37°C for 7 d (N = 3). ^89^Zr-DFO-nimotuzumab was added to 0.9% NaCl or human plasma to make a final concentration of 18 MBq/mL. An aliquot at different time points and up to 7 days were drawn and analyzed for radiochemical purity with SEC-HPLC. An aliquot of ^89^Zr-DFO-nimotuzumab (triplicates) stored at room temperature and 4°C was determined at 2, 6, 16, 24, and 48 h post incubation and purification by SEC-HPLC.

### Radioligand saturation binding assay and immunoreactive fraction determination

The binding of ^89^Zr-DFO-nimotuzumab to EGFR-positive DLD-1 cells was determined using a saturation radioligand binding assay by incubating increasing concentrations of radio-immunoconjugates (0.2 to 95 nmol/L in 100 μL of PBS) for 4 h at 4°C with 200,000 cells. Non-specific binding was determined in a similar assay but in the presence of a 50-fold mole excess of nimotuzumab (relative to the highest concentration of the radio-immunoconjugates, i.e. 4,500 nmol/L). A non-linear regression analysis with One Site - Total and Nonspecific binding equation was used to determine the K_D_ and the binding potential (Bmax) from which the number of receptors per cell was determined (GraphPad Prism V5.02, 2008). Immunoreactivity fractions of ^89^Zr-DFO-nimotuzumab were determined as described by Lindmo *et al*. [22] using DLD-1 cells.

### Pharmacokinetics and dosimetry estimation

Female athymic CD-1 mice (N = 4) were injected intravenously via the tail vein with 6.5±0.0.1 (specific activity 0.38 MBq/μg) ^89^Zr-DFO-nimotuzumab in PBS. Blood samples were collected from the saphenous vein at different time points (0.08–144 h) in a heparinized capillary tube and the blood radioactivity was expressed as the percentage of injected dose/mL (% ID/mL). Pharmacokinetic parameters, including the distribution and elimination half-lives (t_1/2_α and t_1/2_β), volume of distribution at steady-state (Vss), and clearance (CL) were estimated by fitting the blood radioactivity versus time curve to a two-compartment model with i.v. bolus input.

Human radiation dosimetry estimates were calculated from animal biodistribution data obtained by the standard method of organ dissection. Animal biodistribution data was obtained using Balb/c (50 males, 50 females) mice injected with 3.2 ± 0.2 MBq of ^89^Zr-DFO-nimotuzumab divided in 10 groups of 10 animals (5 females/5 males). Animals were euthanized and organs were harvested in groups of five at the following time points: 1, 4, 12, 24, 48, 72, 96, 120, 144, and 168 hours post injection (hp.i). The following organs were harvested, weighed, and counted for radio-activity in a gamma counter: blood, lungs, liver, spleen, kidneys, bladder, muscle, heart, brain, bones, red marrow, testes, adrenals, pancreas, uterus, ovaries, stomach, small intestine, and large intestine. Radioactivity in organs was expressed as % injected activity (%IA) or %IA per gram (% IA/g). For each organ or tissue, the effective data (non-decay-corrected % IA/g) were plotted against sampling time, and used to obtain an estimate of the microcurie-hours per microcurie administered, represented by the area under the time-activity function integrated to infinity (complete decay) of the ^89^Zr. Absorbed doses in units of cGy (centigray) per milicurie (cGy/mCi) Zr-89 administered were calculated. Mouse organ doses were extrapolated hypothetically to the human and recalculated by recalculating the residence times for the human model from the mouse model using OLINDA1.1 [17, 23].

### Single dose toxicity study of ^89^Zr-DFO-nimotuzumab in mice

Acute (Day-2: considering Day 1 as the day of injection) and delayed (Day-14) toxicity studies was carried out in male and female (n = 5 males + 5 females), six week old Balb/C mice (Charles Rivers). Mice were monitored for weight and behavior changes. The vehicle for injection was PBS as it was used in the final preparation of ^89^Zr-DFO-nimotuzumab. 74 MBq (10 mg) of immunoconjugates was proposed maximum dosage for human use. 100 mice were divided in to five groups: 20 mice were used for base line study, 40 mice (control group) were treated with vehicle (PBS). 20 of these were sacrificed as a control of the acute toxicity study on Day-2 and 20 mice were sacrificed as a control of the delayed toxicity study on Day-14. 40 mice (treatment group) were injected with 3.2 MBq, 0.065 mg (which corresponds to 168-fold excess radioactivity dose and 25-fold excess mass dose of ^89^Zr-nimotuzumab that is projected for human studies on a weight/weight basis). 20 of these were sacrificed on Day-2 for the acute toxicity study and 20 of them were sacrificed for delayed toxicity study on Day-14.

All animals in this study were observed regularly for signs of mortality, morbidity, injury, and availability of food and water. Individual body weights were determined and recorded during the quarantine period and (every other day) experimental period. Blood was collected via cardiac puncture for hematology and clinical chemistry. A complete whole blood and plasma analyses were done. Mice were sacrificed on, day 2, or day 14 and organs of interest (kidneys, spleen, liver, bone, heart, lungs, brain, and testes/uterus) were harvested and stored in 10% formaldehyde solution. They were further processed and examined for histopathological analyses.

### Biodistribution and microPET imaging in tumor bearing mice

Biodistribution studies of ^89^Zr-DFO-nimotuzumab was performed in mice bearing EGFR-positive MDA-MB-468, DLD-1, and EGFR-negative MDA-MB-435 xenografts. Female CD-1 nude mice (4 to 6 weeks old) were obtained from Charles River and inoculated subcutaneously with 10^6^ cells on the right flank. Mice (n = 4 per time point) were injected with 10 ±1 MBq 10 μg ^89^Zr-DFO-nimotuzumab injection via tail vein and sacrificed at 4, 24, 72, 120, 144, or 168 h post injection. Blood and major organs were collected and weighed. The radioactivity in blood and organs was counted using an automated γ-counter and expressed as a percentage of injected activity per gram of organ weight (% IA/g).

Tumor bearing mice were injected via a tail vein with 10-12 MBq 10 – 12 μg of ^89^Zr-DFO-nimotuzumab. PET/CT images were acquired at 4, 24, 48, 72, 96, 120, 144, and 168 hp.i using the Vector4CT scanner (MILabs B.V., Utrecht,) PET scans were acquired in a list-mode data format with an high-energy ultra-high resolution (HE-UHR-1.0 mm) mouse/rat pinhole collimator. Corresponding CT scans were acquired with a tube setting of 50 kV and 480 μA. Images were reconstructed using a pixel-based order-subset expectation maximization (POS-EM) algorithm that included resolution, recovery and compensation for distance-dependent pinhole sensitivity and were registered on CT and quantified using PMOD 3.8 software (PMOD, Switzerland). Tracer uptake was expressed as percentage injected activity (% IA) per cc of tissue volume (% IA/cc). All quantification data was reported as mean ± standard deviation within one animal study group.

### Statistical analysis

All data was expressed as the mean ± Stdev of at least 3 independent experiments. Statistical significance between groups was assessed using a two-tailed Student's *t-*test or analysis of variance (ANOVA) with Bonferoni post hoc test. All graphs were prepared and analysed using GraphPad Prism (version 5; GraphPad, La Jolla, CA).

## SUPPLEMENTARY MATERIALS FIGURES AND TABLES





## Abbreviations

PET: Positron Emission Tomography
EGFR: Epidermal Growth Factor Receptor I
DFO: Desferoxamine
mSv: miliSievert
MBq: mega Becquerel
mCi: microCurie
Zr: Zirconium
rem: Roentgen Equivalent Man
IHC: Immunohistochemistry
FISH: Fluorescence In Situ Hybridization
%IA: Percent Injected Activity
%IA/g: per gram
qPCR: Quantitative Polymerase Chain Reaction
HE-UHR: High-Energy Ultra-High Resolution
GMP: Good Manufacturing Practice
cGy: centiGray
SEC: Size Exclusion Column
h.p.i: hour post injection
