# Supplementary material for: 89Zr-nimotuzumab for immunoPET imaging of epidermal growth factor receptor I
Source: Oncotarget. 2018 Mar 30;9(24):17117–32. doi: 10.18632/oncotarget.24965 (PMC5908310; doi:10.18632/oncotarget.24965)
Supplement: Supplementary file 1 [file oncotarget-09-17117-s001.pdf]

# $^{89}$zr-nimotuzumab for immunoPET imaging of epidermal growth factor receptor I

## SUPPLEMENTARY MATERIALS

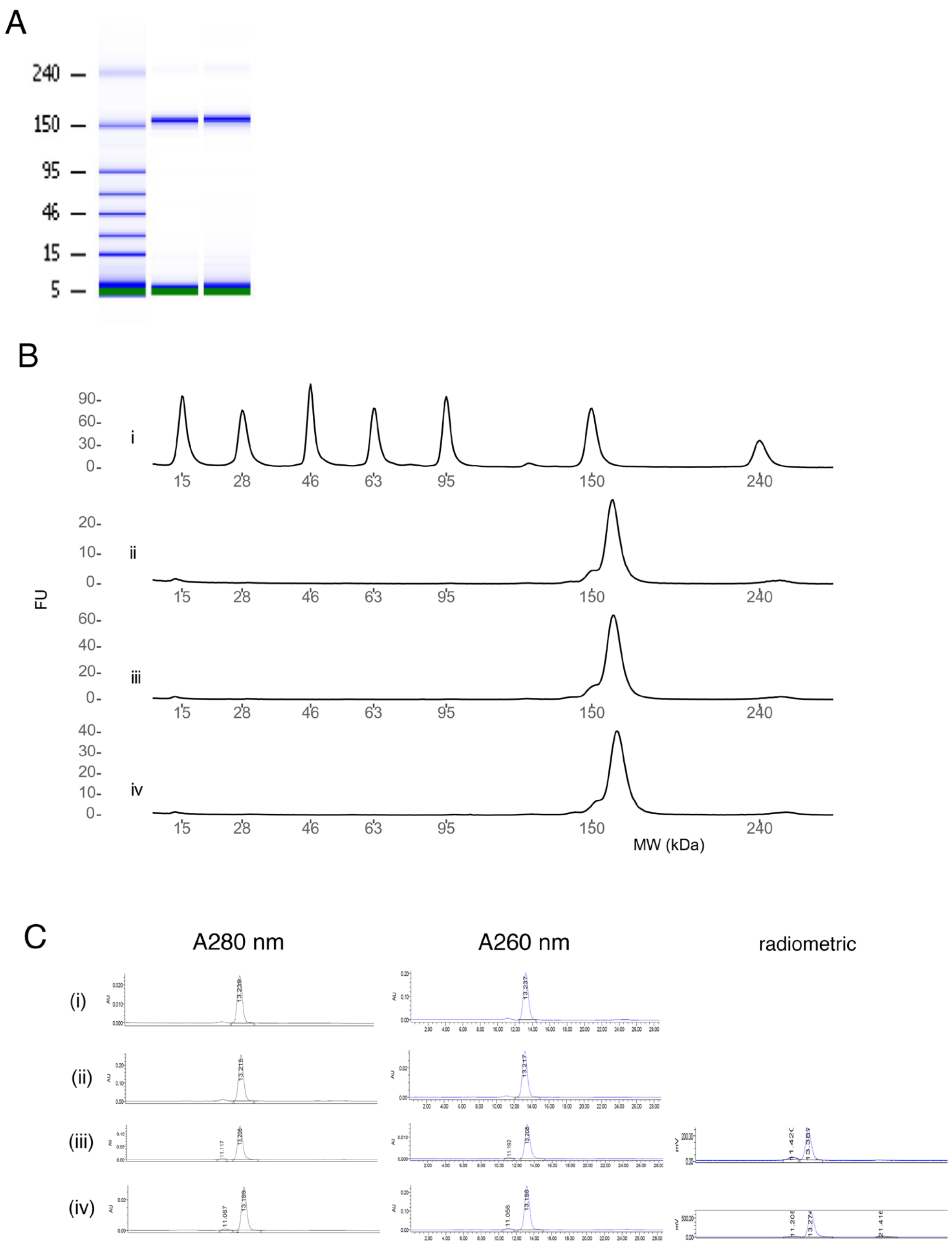


**Supplementary Figure 1: (A and B)** Bioanalyzer ladder (A) and chromatograms (B) of ladder (i) nimotuzumab (ii) DFO-nimotuzumab, (iii) DFO-nimotuzumab (iv). Samples were >95% pure with molecular weight of 161.5 kDa (nimotuzumab) and 162.7 ± 0.9 (DFO-nimotuzumab). **(C)** Representative size exclusion (SEC) HPLC chromatograms showing stability of $^{89}$Zr-DFO-nimotuzumab. UV channel (280 and 220nm) and radiometric channels are shown.

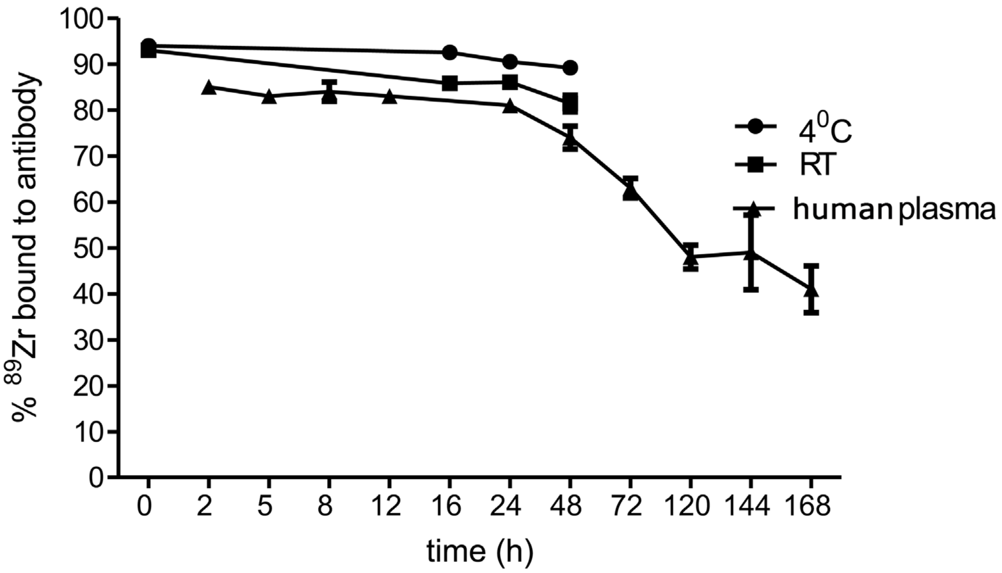


**Supplementary Figure 2: Stability studies of $^{89}$Zr-DFO-nimotuzumab at room temperature (RT), 4°C and in human plasma at 37°C.**

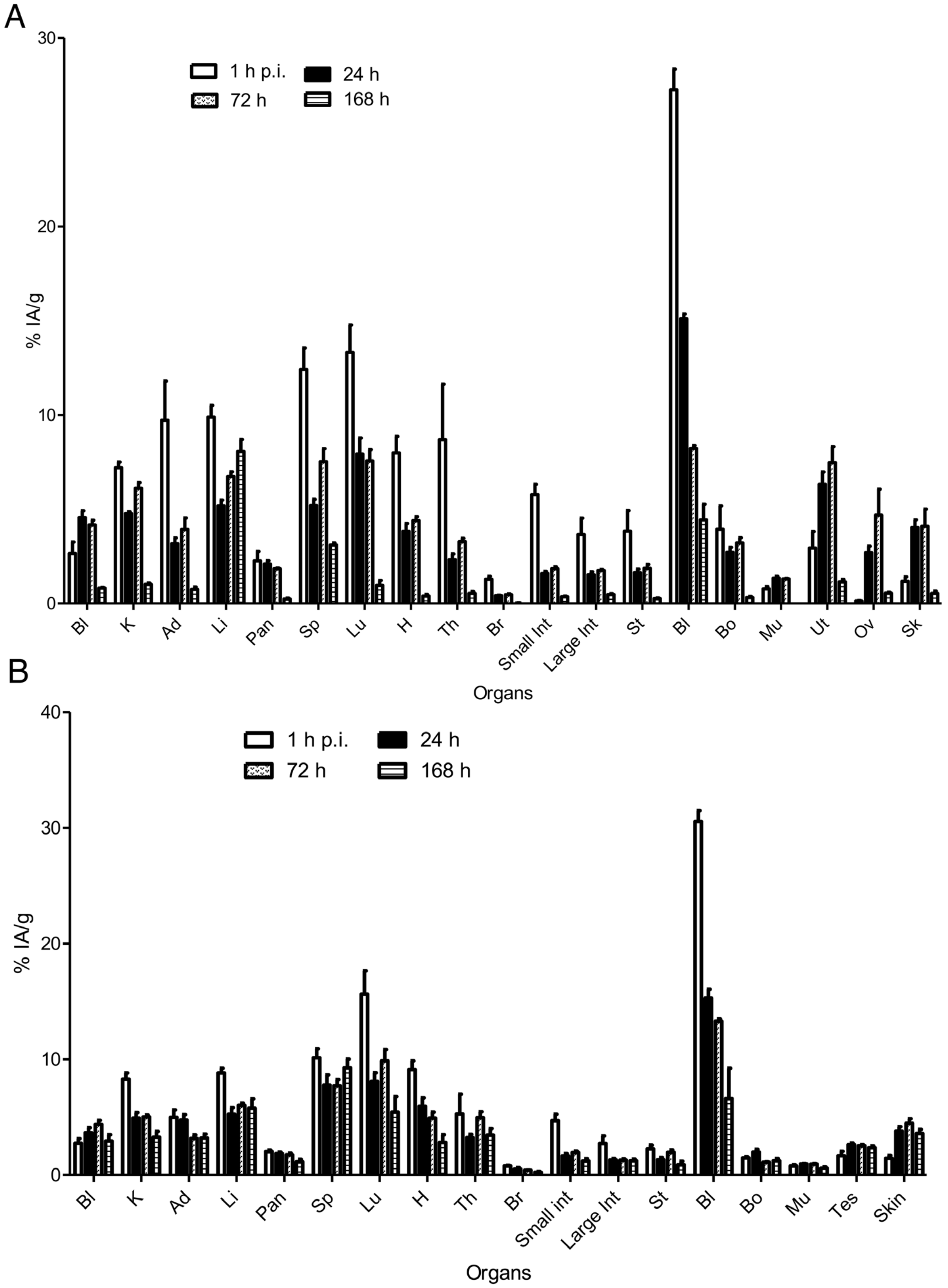


**Supplementary Figure 3: (A and B)** Representative biodistribution in normal balb-C female (A) and male (B) mice following injection of 10 MBq 10 µg of $^{89}$Zr-DFO-nimotuzumab via a tail vein. Groups of mice (n = 4) were sacrificed at 1, 4, 12, 24, 48, 72, 96, 120, 144, and 168 hours post injection (h p.i.). Representative biodistribution at 1, 24, 72 and 168 h p.i. is shown. Bl: bladder; K: kidneys, Ad: adrenals: Li: liver; Pa: pancreas; Sp: spleen; Lu: lungs, H: heart; Th: thymus; Br: brain; Small Int: small intestines; Large Int: large intestines; St: stomach; Bl: blood; Bo: bone; Mu: muscle; Tes: testes; Sk: skin; Ut: uterus; Ov: ovaries.

**Supplementary Table 1: Stability of $^{89}$Zr-DFO-nimotuzumab by HPLC**

| Time (h) | Start (n=3) | 16 h | 24 h | 48 h |
|---|---|---|---|---|
| 4°C | 94.0 ± 0.9 | 92.5 ± 1.5 | 90.5 ± 0.7 | 89.2 ± 1.0 |
| RT | 93.0 ± 1.1 | 85.8 ± 1.3 | 86.1 ± 1.6 | 81.4 ± 1.8 |

**Supplementary Table 2: Residence time of $^{89}$Zr-DFO-nimotuzumab in different organs.**

**See Supplementary File 1**
