# Supplementary material for: 89Zr-nimotuzumab for immunoPET imaging of epidermal growth factor receptor I
Source: Oncotarget. 2018 Mar 30;9(24):17117–32. doi: 10.18632/oncotarget.24965 (PMC5908310; doi:10.18632/oncotarget.24965)
Supplement: Supplementary file 2 [file oncotarget-09-17117-s002.docx]

**Supplementary Table 2: Residence time of ^89^Zr-DFO-nimotuzumab in different organs**

| **Females** | **Mouse**  **Organ** | **Average**  **Weight (g)** | **Residence**  **Time (g^-1^) in the Mouse** | **Residence**  **Time for the Whole Mouse Organ (hrs)** | **Human**  **Model Organ Weight (g)** | **Inferred**  **Human Residence**  **Time (hrs)** |
| --- | --- | --- | --- | --- | --- | --- |
|  |  |  |  |  |  |  |
|  | Bladder | 0.0275 | 3.47376 | 0.095407 | 47.6 | 6.77E-05 |
|  | Kidneys | 0.2584 | 5.15914 | 1.333008 | 299 | 0.0558923 |
|  | Adrenals | 0.0146 | 2.67072 | 0.039057 | 16.3 | 5.053E-06 |
|  | Liver | 0.9087 | 5.59064 | 5.080013 | 1910 | 4.7851333 |
|  | Pancreas | 0.1353 | 1.57192 | 0.212634 | 94.3 | 0.0014721 |
|  | Spleen | 0.0855 | 5.29065 | 0.45215 | 183 | 0.0038379 |
|  | Lungs | 0.1247 | 5.93337 | 0.740164 | 1000 | 0.0501126 |
|  | Heart | 0.0842 | 3.55415 | 0.299174 | 316 | 0.0043191 |
|  | Thymus | 0.0677 | 2.24277 | 0.151741 | 20.9 | 0.0001165 |
|  | Brain | 0.3841 | 0.239909 | 0.092146 | 1420 | 0.0272762 |
|  | Intestines | 0.8330 | 0.877352 | 0.730832 | 677 | 0.2236881 |
|  | Large Intestine | 0.6126 | 1.14518 | 0.701512 | 167 | 0.0389498 |
|  | Stomach | 0.2468 | 1.52202 | 0.375689 | 154 | 0.0077509 |
|  | Bone | 0.0473 | 3.60053 | 0.170319 | 120 | 0.0005247 |
|  | Marrow | 0.0047 | 3.00281 | 0.014166 | 1120 | 4.062E-05 |
|  | Muscle | 0.1751 | 1.09905 | 0.192411 | 28000 | 0.5119074 |
|  | Uterus | 0.1139 | 6.04402 | 0.688438 | 79 | 0.0033622 |
|  | Ovaries | 0.2903 | 3.82845 | 1.111514 | 8.71 | 0.0015255 |
|  | Skin | 0.3691 | 3.45424 | 1.275029 | 3010 | 0.7688589 |
|  | Remainder | 4.5550 | 0.2476 | 1.127818 | 13428 | 37.439603 |
|  |  |  |  |  |  |  |
| **Males** | **Mouse**  **Organ** | **Average**  **Weight (g)** | **Residence**  **Time (g^-1^) in the Mouse** | **Residence**  **Time for the Whole Mouse Organ (hrs)** | **Human**  **Model Organ Weight (g)** | **Inferred**  **Human Residence**  **Time (hrs)** |
|  | Bladder | 0.0216 | 4.00894 | 0.086433 | 47.6 | 4.814E-05 |
|  | Kidneys | 0.3535 | 4.57192 | 1.616357 | 299 | 0.0927341 |
|  | Adrenals | 0.0217 | 3.60363 | 0.078141 | 16.3 | 1.499E-05 |
|  | Liver | 1.0849 | 6.03315 | 6.545364 | 1910 | 7.3612135 |
|  | Pancreas | 0.1522 | 1.62595 | 0.24747 | 94.3 | 0.0019277 |
|  | Spleen | 0.0833 | 8.75611 | 0.729734 | 183 | 0.0060403 |
|  | Lungs | 0.1415 | 7.99352 | 1.131243 | 1000 | 0.0868893 |
|  | Heart | 0.1152 | 4.80928 | 0.554125 | 316 | 0.01095 |
|  | Thymus | 0.0021 | 4.17267 | 0.008951 | 20.9 | 2.178E-07 |
|  | Brain | 0.4310 | 0.449856 | 0.193888 | 1420 | 0.0644034 |
|  | Intestines | 0.9096 | 0.594188 | 0.540462 | 677 | 0.1806287 |
|  | Large Intestine | 0.6942 | 1.21446 | 0.843054 | 167 | 0.053044 |
|  | Stomach | 0.2764 | 1.17319 | 0.324305 | 154 | 0.0074929 |
|  | Bone | 0.0543 | 1.34669 | 0.073186 | 120 | 0.000259 |
|  | Marrow | 0.0048 | 2.12904 | 0.010233 | 1120 | 2.99E-05 |
|  | Muscle | 0.1573 | 0.998622 | 0.157063 | 28000 | 0.3754038 |
|  | Testes | 0.1464 | 2.56521 | 0.375454 | 39.1 | 0.0011662 |
|  | Skin | 0.3213 | 4.00321 | 1.286375 | 3010 | 0.6752835 |
|  | Remainder | 4.5550 | 0.2476 | 1.127818 | 13428 | 37.439603 |
